# A new perspective on proteinuria and drug therapy for diabetic kidney disease

**DOI:** 10.3389/fphar.2024.1349022

**Published:** 2024-07-31

**Authors:** Ruimin Zhang, Qian Wang, Yaqing Li, Qihu Li, Xuefeng Zhou, Xiangmei Chen, Zheyi Dong

**Affiliations:** ^1^ Chengdu University of Traditional Chinese Medicine, Chengdu, China; ^2^ Department of Nephrology, First Medical Center of Chinese PLA General Hospital, National Key Laboratory of Kidney Diseases, National Clinical Research Center for Kidney Diseases, Beijing Key Laboratory of Kidney Diseases Research, Beijing, China

**Keywords:** diabetic kidney disease, proteinuria, pathophysiological mechanism, drug therapy, Chinese patent medicine

## Abstract

Diabetic kidney disease (DKD) is one of the leading causes of end-stage renal disease worldwide and significantly increases the risk of premature death due to cardiovascular diseases. Elevated urinary albumin levels are an important clinical feature of DKD. Effective control of albuminuria not only delays glomerular filtration rate decline but also markedly reduces cardiovascular disease risk and all-cause mortality. New drugs for treating DKD proteinuria, including sodium-glucose cotransporter two inhibitors, mineralocorticoid receptor antagonists, and endothelin receptor antagonists, have shown significant efficacy. Auxiliary treatment with proprietary Chinese medicine has also yielded promising results; however, it also faces a broader scope for development. The mechanisms by which these drugs treat albuminuria in patients with DKD should be described more thoroughly. The positive effects of combination therapy with two or more drugs in reducing albuminuria and protecting the kidneys warrant further investigation. Therefore, this review explores the pathophysiological mechanism of albuminuria in patients with DKD, the value of clinical diagnosis and prognosis, new progress and mechanisms of treatment, and multidrug therapy in patients who have type 2 diabetic kidney disease, providing a new perspective on the clinical diagnosis and treatment of DKD.

## 1 Introduction

Diabetic kidney disease (DKD) is one of the leading chronic microvascular complications in patients who have diabetes. The clinical manifestations include persistently increased urinary albumin levels and/or glomerular filtration rate (GFR) progressively declining. DKD is the leading cause of end-stage kidney disease worldwide, and 30%–50% of end-stage renal disease (ESRD) cases globally are caused by DKD (Lin et al., 2018); DKD accounts for approximately 50% of cases of ESRD on dialysis or kidney transplantation in the United States (DeFronzo et al., 2021). DKD also significantly increases the risk of cardiovascular disease in patients with diabetes and is an important risk factor for premature death (Johansen et al., 2021).

Proteinuria in patients with DKD is primarily albuminuria, which has long been considered an important clinical feature of DKD. Notably, new therapeutic drugs for albuminuria in DKD are being developed and are widely used. Normoalbuminuric diabetic kidney disease (NADKD) is also gradually being recognized, and the timing of albuminuria control in patients with DKD has become more challenging; therefore, albuminuria in patients with DKD should be re-examined. This paper summarizes the pathophysiological mechanism of albuminuria in patients with DKD, the value of clinical diagnosis and prognosis, new progress and mechanisms of treatment, and multidrug therapy in patients with type 2 diabetic kidney disease (T2DKD), providing a new perspective on the clinical diagnosis and treatment of DKD.

## 2 Pathophysiological mechanism of albuminuria in DKD

Albuminuria is a prominent feature of DKD, which not only reflects damage to the glomerular filtration barrier but is also affected by glomerular hyperfiltration and renal tubular reabsorption. The presence of albuminuria indicates the excretion of albumin from the glomeruli and the reabsorption capacity of renal tubules for albumin (Heyman et al., 2022).

On the glomerular scale, hyperfiltration, endothelial dysfunction, basement membrane thickening, podocyte foot process fusion or detachment, glomerulosclerosis, and arteriolar hyalinization may lead to proteinuria (Heyman et al., 2022). Recent studies have demonstrated that both podocyte DNA double-strand breaks and glomerular DNA methylation are associated with the severity of albuminuria (Yoshimoto et al., 2023).

In the renal tubules, cubilin/megalin downregulation, tubular inflammation, atrophy, and reduced reabsorption of amino acids and proteins by the proximal tubules play a role in the progression of DKD exacerbated by proteinuria (Li et al., 2022; Heyman et al., 2022). The mechanism underlying renal tubular proteinuria is primarily related to damage to the structure and function of the proximal tubules. Renal tubular epithelial cells are located in chronic high-glucose or hypoxia-ischemic environments and exhibit abnormal metabolism and hemodynamics. Additionally, they express advanced glycation end-product (AGE), reactive oxygen species, and other metabolites. Through a series of kinase cascades, they lead to extracellular matrix accumulation or macrophage infiltration to induce inflammation, eventually resulting in renal tubulointerstitial fibrosis and tubular injury, affecting renal tubular reabsorption function. This leads to renal tubular proteinuria development.

**FIGURE 1:**
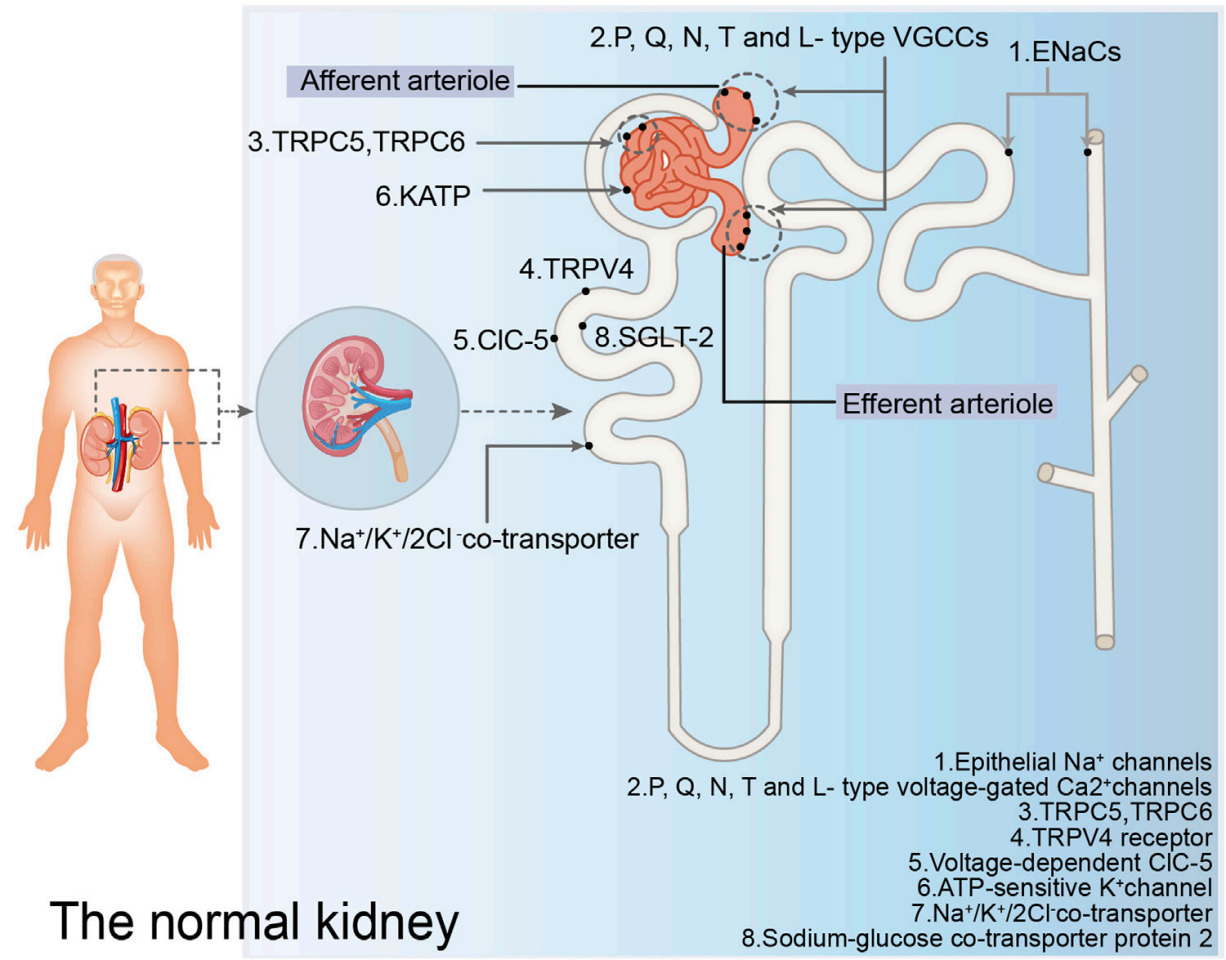
Ion channels and transporters mentioned in the normal kidney. The figure illustrates the ion channels and ion transporters in the glomerulus, proximal convoluted tubules, distal convoluted tubules, and collecting ducts discussed in this paper that play a role in regulating proteinuria. EnaCs: epithelial Na^+^ channels; P, Q, N, T and L-type VGCCs: P, Q, N, T and L-type voltage-gated Ca^2+^ channels; TRPV4: TRPV4 receptor; ClC-5: voltage-dependent ClC-5; KATP: ATP-sensitive K^+^ channel; SGLT-2: sodium-glucose co-transporter 2.

Ion channels and transporters play important roles in the pathogenesis of proteinuria (Liu et al., 2023) (Figure 1). Regarding ion channels, blocking the epithelial Na^+^ ion channels located in the epithelial cells of the distal convoluting tubules and collecting ducts can prevent Na^+^ reabsorption, restore the balance of tubuloglomerular feedback, decrease Na^+^ retention, ameliorate glomerular hyperfiltration, and reduce glomerular albumin leakage (Shen et al., 2021; Xiao et al., 2022). Blocking the P/Q-, N-, T-, and L-type voltage-gated Ca^2+^ channels located in the arterioles of glomeruli can significantly restore the expression of podocyte-related proteins such as nephrin and podocin, protect podocytes, and reduce glomerular proteinuria (Tamargo and Ruilope, 2016). Moreover, blocking the TRPC5 (Zhou et al., 2017) and TRPC6 (Hall et al., 2019) ion channels located in podocytes can protect the podocyte cytoskeletal hub structure, actin cytoskeleton, and reduce glomerular proteinuria. Blocking Ca^2+^ -activated K^+^ channels located in glomeruli and some renal tubules, as well as inhibiting Inflammatory factor-related signaling pathways such as growth factor-beta 1 (TGF-β1) signaling pathway and the expression of inflammatory markers, can inhibit extracellular matrix deposition in the kidney and mitigate kidney injury (Huang et al., 2018). The activation of TRPV4 in proximal tubular epithelial cells can stimulate albumin endocytosis of tubular epithelial cells and reduce proteinuria (Gualdani et al., 2020). This activation also protects the expression of ClC-5, protects the reabsorption of albumin by the proximal tubule, stimulates albumin endocytosis, and reduces proteinuria (Jentsch and Pusch, 2018). Furthermore, TRPV4 activation stimulates ATP-sensitive K^+^ channels located in the mitochondrial membrane of podocytes, inhibits the production of reactive oxygen species and podocyte injury, and reduces glomerular proteinuria (Tinker et al., 2018). The administration of N-acetylcysteine has been shown to mitigate renal lesions in elderly rats, upregulate Na^+^/K^+^/2Cl^−^ cotransporters in the proximal tubular brush border membrane, and alleviate proteinuria (Shimizu et al., 2013). The mechanism by which N-acetylcysteine alleviates proteinuria in aging rats may involve simultaneous protection of glomerular and tubular functions. It safeguards glomerular filtration function, reduces glomerular albumin leakage, thereby decreasing albumin reabsorption by renal tubular epithelial cells and reducing renal tubulointerstitial damage. Additionally, it mitigates oxygen free radicals induced renal tubular epithelial cell damage, preserves the expression of Na^+^/K^+^/2Cl^−^cotransporters, and protects albumin endocytosis. The deeper the pathophysiological mechanism of DKD, the more precise the treatment for DKD.

**FIGURE 2:**
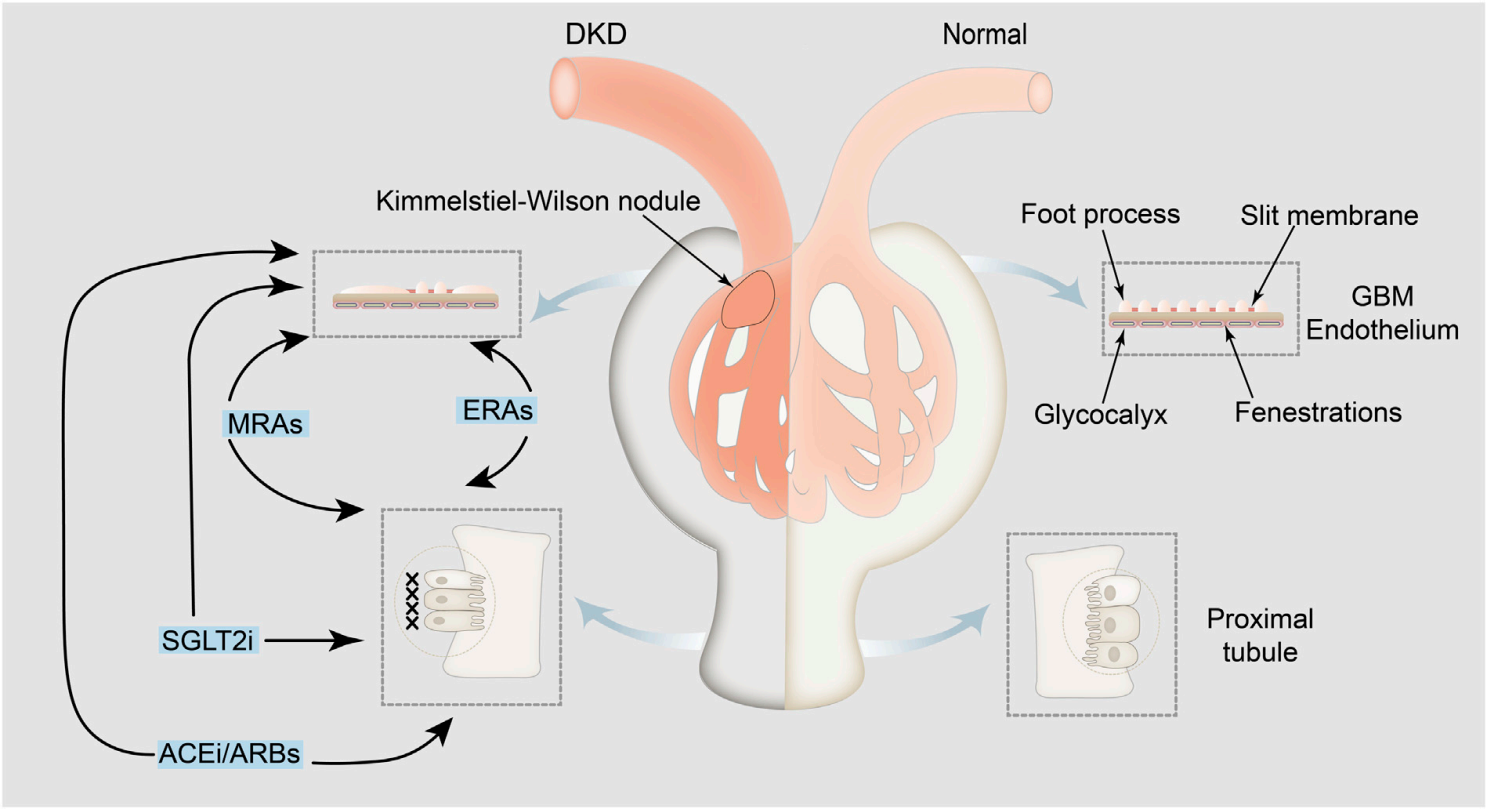
Pathophysiological mechanisms underlying albuminuria in patients with DKD and related drugs. Albuminuria is indicative of albumin leakage from the glomerulus, with the exclusion of albumin reuptake by the renal tubules. Medications aimed at treating albuminuria, such as angiotensin-converting-enzyme inhibitors/angiotensin receptor blockers (the first and leading medications that reduce proteinuria), SGLT2 inhibitors, nonsteroidal mineralocorticoid receptor antagonists, and endothelin receptor antagonists, directly or indirectly impact both glomerular albumin leakage and tubular reabsorption of albumin, thereby mitigating albuminuria. The left panel shows pathological glomerular filtration membranes, atrophic renal tubular epithelial cells and interstitial fibrosis and the mechanism of drug action. The right panel shows the physiological mechanism of the kidney in a normal person. GBM: glomerular basement membrane; SGLT2i: SGLT2 inhibitors; MRAs: nonsteroidal mineralocorticoid receptor antagonists; ERAs: endothelin receptor antagonists; ACEi: angiotensin-converting-enzyme inhibitors; ARBs: angiotensin receptor blockers.

Overall, this study summarizes the pathophysiological mechanisms underlying albuminuria in patients with DKD (Figure 2).

## 3 Role of albuminuria in the diagnosis and prognosis of DKD

According to the degree of proteinuria in patients with DKD, they are categorized into microalbuminuria [urinary albumin-to-creatinine ratio (UACR) 30–299 mg/g] and macroalbuminuria (UACR ≥300 mg/g). Microalbuminuria is used to indicate the possibility of chronic kidney disease (CKD) in patients (Andrassy, 2013). Proteinuria is an important risk factor for renal prognosis and is not the only diagnostic criterion for DKD. An artificial intelligence system for detecting urinary microalbuminuria is expected to monitor the progression of diabetic nephropathy (DN) based on laboratory examinations (Lin et al., 2022). Studies have confirmed that in patients with diabetes with any level of eGFR, the presence of proteinuria is associated with the risk of cardiovascular disease, CKD progression, and death (van der Velde et al., 2011; Kovesdy et al., 2013). Therefore, for patients with diabetes with macroalbuminuria, the treatment goal is to reduce urinary albumin by ≥ 30% to delay CKD progression. For patients with diabetes with microalbuminuria, the risk of progressin to macroalbuminuria should be reduced (American Diabetes Association Professional Practice Committee, 2024).

According to the concept of proteinuria defined by the National Kidney Foundation and the US Food and Drug Administration in 2009 (Levey et al., 2009), NADKD (UACR <30 mg/g) should be further refined to UACR <10 mg/g and UACR 10–29 mg/g groups. A UACR of <10 mg/g is the normal urine albumin content, and a UACR in the range of 10–29 mg/g exceeds the normal value (An et al., 2022). However, a better understanding of NADKD may facilitate its early diagnosis.

## 4 Timing of control of albuminuria in patients with DKD

The phenotype of normoalbuminuric renal failure may be linked to efficient tubular reabsorption of albumin, and increased albuminuria may further indicate deteriorating tubular reuptake (Heyman et al., 2022). Some studies have found that some patients already have ESRD without proteinuria (Oshima et al., 2021), and the specific underlying mechanism remains unclear, which may be related to hypertension, unrecovered acute kidney injury, and renal vascular disease. Data from an autopsy study of 105 community patients with normoalbuminuric diabetes in Japan, published in 2021, showed that advanced DN diagnosed using pathological analysis accounted for approximately 50% of cases. This suggests that the absence of detectable albuminuria does not rule out the presence of DKD. A Spanish autopsy study in 2022 with a small sample of 21 patients reached similar conclusions (D’Marco et al., 2022). The time at which patients with type 2 diabetes (T2D) develop into DKD still remains unknown; however, these findings may lead to a breakthrough in future clinical practice regarding whether early treatment with renin-angiotensin-system inhibitors (RASi) or SGLT2 inhibitors (SGLT2i) should be initiated before microalbuminuria develops in patients with diabetes. Future noninvasive diagnostic techniques may help identify patients with cryptic DKD who may benefit from early treatment (Smith et al., 2021; Jiang et al., 2023). Therefore, more randomized controlled trials (RCTs) and real-world studies are needed to help clinicians determine the appropriate time to control albuminuria in patients who suffer DKD.

## 5 Treatment mechanism and progress of albuminuria in patients with DKD

The rationale behind the use of RASi and SGLT2i in clinical treatment of albuminuria in DKD patients primarily involves their ability to reduce glomerular hyperfiltration by modulating hemodynamics, thereby decreasing glomerular pressure and albuminuria (Heyman et al., 2022). The RENAAL study, a previous large RCT, confirmed the renoprotective effect of reducing proteinuria in patients with T2D (Brenner et al., 2001). Patients receiving losartan showed a 26% decrease in proteinuria within 3 months, a further decrease of more than 40% within 3 years, and a significant improvement in renal survival. The other RASi were equally effective in reducing the progression of proteinuria and renal insufficiency. As the first landmark drug for DKD treatment, it has been used in clinical practice for more than 20 years. Both the 2024 American Diabetes Association (ADA) guidelines (American Diabetes Association Professional Practice Committee, 2024) and the 2022 Kidney Disease: Improving Global Outcome (KDIGO) guidelines (KDIGO, 2022) recommend the use of RASi in treating microalbuminuria and macroalbuminuria in patients with DKD; recommend the combination of SGLT2i for patients with T2D and CKD who have a UACR ≥ 200 mg/g creatinine and eGFR ≥ 20 mL/min·1.73 m^2^, supported by evidence level A.

### 5.1 Angiotensin-converting-enzyme inhibitors/angiotensin receptor blockers

Angiotensin II (AngⅡ) is a key mediator of the renin-angiotensin system (RAS). Upon RAS activation, AngⅡ induces various effects, including systemic arteriolar constriction, increased secretion of antidiuretic hormone, promotion of water and sodium reabsorption, and increased secretion of aldosterone (Ksiazek et al., 2024). Angiotensin-converting-enzyme inhibitors (ACEi), as direct inhibitors of angiotensin-converting-enzyme (ACE), inhibit the production of AngⅡ. Angiotensin receptor blockers (ARB) prevent the binding of AngⅡ to its type 1 receptor and thereby mitigate the aforementioned effects. The mechanisms by which ACEi/ARB reduces glomerular proteinuria primarily involve a decrease in glomerular pressure through relative relaxation of the efferent arterioles (Lovshin et al., 2018) and a reduction in glomerular albumin leakage by diminishing the surface area of the filtration barrier (glomerular contraction) (Nistala et al., 2021). In DKD, the N-domain of ACE drives renal inflammation (Crowley et al., 2024), while AngⅡ activates angiotensin type 1 receptor to promote inflammation (Cantero-Navarro et al., 2021); however, ACEi/ARB exerts an anti-inflammatory effect and reduces proteinuria.

The commonly used ACEi/ARB classes in clinical practice include captopril, valsartan, olmesartan, losartan, etc. The Chinese guidelines for the clinical diagnosis and treatment of DKD (Nephrology, 2021) also recommend that ACEi/ARB drugs are the first-line therapy to reduce proteinuria in patients with T2DKD with microalbuminuria or macroalbuminuria. However, the combination of ACEi and ARB is not recommended.

### 5.2 SGLT2 inhibitors

The main mechanism of SGLT2i in treating albuminuria in patients with DKD is to restore tubuloglomerular feedback and alter glomerular hemodynamics, thereby reducing trans-glomerular pressure and glomerular protein leakage (Muskiet et al., 2019; González-Albarrán et al., 2022). Since the mechanism of action of SGLT2i is independent of insulin, it is still functional in patients with type 1 diabetic kidney disease (Warren et al., 2019; Hartman et al., 2020; Wang et al., 2021). SGLT2, located in the proximal convoluted tubules (PCT) (Kogot-Levin et al., 2020), is upregulated in the state of diabetes, leading to increased reabsorption of sodium and glucose by PCT. This results in reduced sodium concentration in the macula densa, suppressing tubuloglomerular feedback and causing dilation of the afferent arterioles. Consequently, there is an increase in trans-glomerular pressure and increased glomerular protein leakage (Heyman et al., 2022). SGLT2i reverse this process caused by SGLT2 upregulation in the diabetic state. SGLT2i induces the production of adenosine, activates the adenosine A1 receptor, increases cytosolic Ca^2+^, and restores glomerulotubular balance, thereby achieving the contraction of afferent arterioles (Kim and Kim, 2022). In patients with T2D receiving RAS blockers, SGLT2i causes dilation of the efferent arterioles upon RAS blockade by increasing adenosine and prostaglandin production (Kim and Kim, 2022). SGLT2i has a renoprotective effect in reducing albuminuria by constricting and dilating afferent and efferent arterioles, respectively, during RAS blockade, lowering intraglomerular pressure and reducing glomerular hyperfiltration. Because SGLT2 co-localizes with sodium-hydrogen exchanger 3 (NHE3) in PCT, SGLT2i blocks NHE3, which makes the natriuretic effect more significant (Salvatore et al., 2022). In clinical and animal experiments, SGLT2i has been shown to increase podocyte autophagy, lower podocyte lipid content, protect podocytes, and reduce proteinuria (DeFronzo et al., 2021; Chen et al., 2022; Durcan et al., 2022). Recent studies have demonstrated that dapagliflozin can delay renal tubulointerstitial fibrosis by inhibiting YAP/TAZ activation (Feng et al., 2023), and block CYP4A/20-HETE signaling to reduce reactive oxygen species and inflammation, thereby decreasing glomerulosclerosis and renal tubulointerstitial fibrosis (Dia et al., 2023). SGLT2i provides novel perspectives for treating glomerular and tubular proteinuria.

Recently, the introduction of SGLT2i has revolutionized the clinical management of T2D, not only effectively improving the cardiovascular outcomes of patients (Caruso and Giorgino, 2022) but also having a renoprotective effect in patients with and without diabetes with moderate and severe renal insufficiency, significantly reducing proteinuria and becoming the second landmark drug for treating DKD (Muskiet et al., 2019; Górriz et al., 2020; Palmer et al., 2021; Forst et al., 2022; Kaze et al., 2022; Luo et al., 2023). Therefore, SGLT2i is recommended to delay CKD progression and reduce the risk of cardiovascular events in patients with CKD with T2D and large albuminuria in recent guidelines. Clinically available SGLT2i mainly include Empagliflozin, Dapagliflozin and Canagliflozin, which have been approved for the treatment of patients with T2D. Compared with SGLT2i drugs, dipeptidyl peptidase-4 inhibitors and glucagon-like peptide-1 agonists have been shown to reduce proteinuria but have not been demonstrated to alleviate the deterioration of renal function (Nicotera et al., 2020; Michos et al., 2023). Encouraging results were expected from the FLOW trial (Rossing et al., 2023) using semaglutide.

### 5.3 Nonsteroidal mineralocorticoid receptor antagonists

The renal protective mechanism of finerenone in patients with DKD is mainly due to its anti-inflammatory and anti-fibrosis effects (Agarwal et al., 2021; Barrera-Chimal et al., 2022a), and it may also achieve anti-albuminuria by improving hemodynamics (Barrera-Chimal et al., 2022a). In patients with DKD, aldosterone activity is increased in the kidney, and mineralocorticoid receptors (MRs) are overactivated, leading to the overexpression of MR-mediated proinflammatory and pro-fibrotic genes in various somatic cells in the kidney, including distal tubular epithelial cells, macula densa, endothelial cells, mesangial cells, podocytes, macrophages, fibroblasts, and other cells, driving renal inflammation and fibrosis (Barrera-Chimal et al., 2022b; Chaudhuri et al., 2022; González-Juanatey et al., 2023; Yao et al., 2023). MR over-activation leads to a series of renal pathological changes, including glomerular basement membrane thickening, podocyte injury, mesangial cell proliferation and apoptosis, mesangial expansion, macrophage infiltration, collagen deposition, glomerulosclerosis, renal tubular epithelial cell atrophy and renal interstitial fibrosis, renal tubular inflammation, endothelial dysfunction, and arteriosclerosis (González-Juanatey et al., 2023; Kim et al., 2023; Lv et al., 2023). These histopathological changes are important mechanisms that lead to albuminuria in patients with DKD. In the cytoplasm, finerenone binds to MR instead of aldosterone, resulting in a conformational change of MR. The MR-finerenone is translocated to the nucleus and binds to the hormone response element, where it cannot transcribe target genes due to the lack of transcription cofactors, leading to the downregulation of proinflammatory and pro-fibrotic genes, such as NF-κB and AP-1, and ultimately reducing renal inflammation and fibrosis (Kim et al., 2023; Ortiz et al., 2023). Furthermore, it alleviates the pathological changes in glomerular and renal tubular damage and improves albuminuria. Regarding hemodynamics, finerenone may improve glomerular hyperfiltration by preventing aldosterone-mediated contraction of efferent arterioles over afferent arterioles, reducing intraglomerular pressure, and achieving anti-albuminuria (Barrera-Chimal et al., 2022a). However, a multicenter, multiracial RCT involving 823 patients with T2D and albuminuria treated with RAS blockade showed no significant association between dose-dependent reductions in UACR with finerenone and reductions in blood pressure or estimated glomerular filtration rate (eGFR) (Bakris et al., 2015), which may indicate that the mechanism by which finerenone reduces albuminuria is hemokinesis-independent (Agarwal et al., 2022a).

Finerenone, which is a nonsteroidal mineralocorticoid antagonist (MRA), is the only nonsteroidal MRA clinically proven to have renal and cardiovascular benefits (Rossing et al., 2022a). It can rapidly reduce proteinuria and provide long-term renal protection in patients with diabetes treated with RASi (Rossing et al., 2022b; Raj, 2022). Additionally, the 2020 KDIGO guidelines (KDIGO, 2020) recommend a combination of ACEi/ARB and MRAs to reduce urinary protein levels in patients with T2DKD. The use of MRAs in treating patients with diabetes with CKD was further improved according to the 2022 KDIGO (KDIGO, 2022) and 2024 ADA guidelines (American Diabetes Association Professional Practice Committee, 2024).

### 5.4 Endothelin receptor antagonists

The expression of endothelin-1 (ET-1) is increased in the kidney of patients with DKD (Kohan and Barton, 2014; Zhang et al., 2023; Martínez-Díaz et al., 2023). ET-1 binds to the endothelin receptor -A (ET-A) to produce pathological effects through various stimulation of kidney histopathological changes, such as damage to the endothelial cell glycocalyx and actin cytoskeleton of podocytes, causing dysfunction of the podocyte membrane, changing the basement membrane, causing mesangial matrix deposition, damaging renal blood vessels, enhancing vascular reactivity and coagulation, leading to renal tubulointerstitial fibrosis, and promoting inflammatory cell infiltration (Kohan and Barton, 2014; Heerspink et al., 2019; Smeijer et al., 2021). ET-A antagonists increase endothelial glycocalyx, reduce glomerular heparanase, protect podocyte actin cytoskeleton and slit diaphragm function, and reduce inflammation, interstitial fibrosis, and extracellular matrix hyperplasia through various mechanisms (Allison, 2016; Garsen et al., 2016). Animal experiments have shown that blocking ETA can reverse the glomerular hyperosmolar state and reduce glomerular albuminuria caused by glomerular injury (Dolinina et al., 2019). Therefore, ET-A antagonists can be used to treat glomerular albuminuria by protecting the glomerular filtration barrier and reversing glomerular hyperfiltration. Additionally, they protect renal tubular epithelial cells, reduce renal tubulointerstitial fibrosis.

Low-dose, short-term treatment with the selective endothelin receptor A antagonist atrasentan has a positive therapeutic effect in reducing albuminuria and improving renal prognosis in some patients with T2DKD without causing significant sodium retention (de Zeeuw et al., 2014; Heerspink et al., 2019; Heerspink et al., 2023), and its clinical application still has broad development prospects. However, the results of the SONAR study showed that the use of atrasentan did not provide cardiovascular protection (Fernandez-Fernandez et al., 2019); therefore, it is necessary to be alert to the occurrence of cardiovascular adverse events during its clinical application. Additionally, the SONAR trial was conducted to ensure safety and introduce strict inclusion and exclusion criteria, excluding patients who were unresponsive to atrasentan (proteinuria reduction ≤ 30%) or intolerant (history of heart failure or peripheral edema). Therefore, it generalizing these results for clinical applications is difficult. Furthermore, unresolved scientific questions remain, such as whether patients with T2DKD who do not respond to atrasentan benefit from SGLT2i-, finerenone-, or insulin-based therapies (Muskiet et al., 2019). However, whether early albuminuria reduction with atrasentan treatment can predict long-term renoprotective effects remains unknown (Heerspink et al., 2021). Therefore, the clinical value of endothelin receptor antagonists (ERA) in patients with T2DKD still needs substantial medical-based evidence.

### 5.5 Chinese patent medicine

#### 5.5.1 Keluoxin capsule

Traditional Chinese medicine involves multiple targets and pathways and can regulate the overall situation of disease treatment. Clinical studies and animal trials have suggested that keluoxin capsules can delay or treat DKD by improving glycolipid metabolism and microcirculation, enhancing histiocyte function, and alleviating renal damage (Premaratne et al., 2015). Keluoxin capsules can improve glycolipid metabolism, regulate tubuloglomerular feedback to reduce kidney tubule injury and renal hypertrophy, and alleviate kidney hyperfiltration (Chunxue et al., 2023). Additionally, Keluoxin capsules lower endothelin, thromboxane B2, atrial natriuretic peptide, and angiotensin (L et al., 2000). Meanwhile, they can also enhance the level of 6-keto-prostaglandin1α, regulate the dynamic balance of the renin-prostacyclin system, and improve hemodynamics and hemorheology and renal microcirculation (L et al., 2000). Simultaneously, Keluoxin capsules can decrease the level of oxidative stress and inflammatory cytokines, probably because they can regulate the JAK/STAT, MAPK, and NF-κB signaling pathways (Kong et al., 2022; Deng et al., 2023; Huimin et al., 2023; XM et al., 2023). Moreover, Keluoxin capsules can regulate podocyte autophagy, alleviate renal damage, and protect the kidney (Yang et al., 2021). Research on the mechanism of action of keluoxin capsules is ongoing. We expect that the mechanism of action will be interpreted more comprehensively.

Keluoxin capsules are composed of Astragali radix, Pseudostellariae radix, Ligustri lucidi fructus, Lycii fructus, Rhei radix et rhizome, and Hirudo. It was the first Chinese patented medicine approved for the treatment of DKD. It can maintain kidney function, reduce the level of urinary protein, delay DKD development, and alleviate related symptoms (Xianhong et al., 2014; Shanshan et al., 2020; Qianqin and Tao, 2022). Keluoxin capsules have been recommended to control proteinuria in patients with DKD according to several guidelines, such as the Guidelines for Diagnosis and Management of Diabetic Kidney Disease with Integrated Traditional Chinese and Western Medicine (Association, 2023) and the Guidelines for the Prevention and Treatment of Type 2 Diabetes Mellitus in China (Society, 2021). A double-blind RCT suggested that keluoxin capsules can prominently reduce the UACR level in patients with early DKD and the ratio of those with deteriorated proteinuria, whose UACR levels continue to rise by 30% (Zhao et al., 2024). According to the study, Keluoxin capsules can also alleviate clinical symptoms, such as fatigue, dry mouth and throat, limb numbness, and pain, aggravated at night, to improve the life quality of patients. The latest meta-analysis, involving 20 studies and 1,500 participants, has suggested that a combination of Keluoxin capsules and Western medicine presents better efficiency than a single use of Western medicine in treating DKD, including improving eGFR and reducing the level of microalbuminuria, urinary albumin excretion rate (UAER), 24-hour urine protein, serum creatinine (Scr), blood urea nitrogen, fasting blood glucose, total cholesterol, triglyceride (TG), and low-density lipoprotein (Wenhua et al., 2022). Regarding safety, no statistically significant differences were found in adverse drug events between the two groups. Pharmacoeconomic research has shown that the combined use of keluoxin capsules and chemical medicine is more economical than chemical medicine alone, considering its current price level (Chang et al., 2022).

#### 5.5.2 Huangkui capsule

A recent animal study has demonstrated that the Huangkui capsule can protect podocytes from doxorubicin (DOX)-induced proteinuria by inhibiting the JAK2/STAT3 and PI3K/Akt pathways and increasing the expression of Nephrin and Podocin in DOX-exposed podocytes (Zhao et al., 2023). Thus, proteinuria was alleviated. Another animal experiment confirmed the advantages of the Huangkui capsule in treating DN from a different perspective, such as regulating the intestinal microbiota and improving metabolite levels in DN (Shi et al., 2023). In terms of reducing renal interstitial fibrosis, animal experiments have demonstrated that Huangkui capsule combined with metformin can effectively improve DN by inhibiting the expression of renal fibrosis-related proteins and blocking Klotho/TGF-β1/p38MAPK signaling pathway in DN rats (Gu et al., 2021). Huangkui capsule inhibited NLRP3 inflammasome activation and TLR4/NF-κB signaling pathway and alleviated epithelial to mesenchymal transition of renal tubules (Han et al., 2019).

Evidence from the real world shows that Huangkui capsules are widely used to treat proteinuria in patients with DKD, with sufficient evidence of clinical efficacy and high frequency of use (Shao et al., 2021; Tan et al., 2023). For example, a multicenter RCT study involving 413 patients with DKD demonstrated that after 24 weeks of treatment, Huangkui capsules combined with irbesartan had significantly greater reductions in UACR, 24-h proteinuria, and urinary protein creatinine ratio than irbesartan alone (*P* < 0.001, *P* = 0.001, and *P* = 0.001, respectively); the overall incidence of adverse events was low and largely similar between treatment groups, with no serious adverse events of reduced liver or renal function (Zhao et al., 2022). A recent meta-analysis of 13 RCTs has shown that Huangkui capsules can effectively reduce 24-hour urinary protein content in patients with CKD. The effectiveness of Huangkui capsules in DKD subgroups should be further clarified; therefore, larger sample sizes and high-quality RCTs in multiple countries are needed to confirm this (Wei et al., 2023).

#### 5.5.3 *Tripterygium* glycosides

The mechanism of action of *T*. *wilfordii* in treating DN has not been fully elucidated. Untargeted metabolomic analysis using ultra-high performance liquid chromatography-quadrupole time-of-flight mass spectrometry revealed that *Tripterygium* improved impaired renal function mainly by promoting TG catabolism in DN mice (Zhang et al., 2022). In China, *T. wilfordii*, combined with Western medicine, is widely used in patients with DKD. Many clinical RCTs have shown that *T. wilfordii* combined with Western medicine can effectively reduce 24-h urinary protein levels and UAERs (Wu et al., 2020; Ma et al., 2021; Xie et al., 2022). However, its clinical application is limited due to the presence of minor side effects that mainly focus on abnormal liver function (Xie et al., 2022). Therefore, to explore its clinical safety, a meta-analysis involving 31 RCTs suggested that the duration of TG-mediated DN treatment should be less than three consecutive months (Li et al., 2021). Therefore, because of the generally low-quality evidence provided by systematic reviews and meta-analyses of *Tripterygium* for the adjuvant treatment of clinical DKD, it still needs to be treated cautiously (Shi et al., 2022). Serious side effects should be carefully monitored, even in courses that are generally considered safe. Large-scale and long-term multicenter studies of *T. wilfordii* combined with Western medicine are expected.

#### 5.5.4 Other Chinese patent medicine

Niaoduqing granules can be used to effectively treat proteinuria in patients with DKD. Through network pharmacology, it has been found that the mechanism of Niaoduqing granules in treating DN proteinuria is to inhibit the activity of the advanced glycation end-product–receptor for advanced glycation end-product (AGE/RAGR) pathway and the overexpression of VEGF-A, ICAM-2, PTGS-5, and ACE in MPC1 cells to prevent podocyte injury in DN (Fang et al., 2023). However, evidence of adverse drug reactions remains unclear (Fu et al., 2023). An ESRD rat model confirmed that Niaoduqing granules improved renal fibrosis and urinary protein excretion by regulating the p38 MAPK/NF-κB signaling pathway (Li et al., 2022). Niaoduqing granule, as an adjuvant therapy for DKD proteinuria, is frequently utilized in conjunction with ACEi/ARB medications in clinical practice. This combination significantly reduces 24-h urinary protein excretion and improves renal function indicators in DKD patients, surpassing the clinical efficacy of Western medicine alone (Zhigang et al., 2020; Gang et al., 2022; Zeqiong and Xu, 2024).

Yishen Huashi granules are widely used to treat proteinuria in patients with DKD, and their therapeutic mechanism is related to the improvement in podocyte injury induced by macrophage-derived exosomes (Liang et al., 2022). Recent animal experiments have shown that Yishenhuashi granules can improve the pathological mechanism of DKD by regulating the “gut-kidney axis” of intestinal flora and serum metabolites and improving the mRNA expression of the kidney (Han et al., 2023). A meta-analysis aimed to assess the clinical efficacy of Yishen Huashi granules in 1,254 patients with DKD. The results showed that compared with conventional western medicine treatment, Yishen Huashi granule significantly reduced UAER, Scr, blood urea nitrogen, total cholesterol, low-density lipoprotein cholesterol and 2-h postprandial glucose in patients with DKD, while increasing serum albumin. However, there were no significant effects on high-density lipoprotein cholesterol, 24-hour urine protein, triglycerides and fasting plasma glucose (Cuiping et al., 2020). Another clinical study by Song (Song et al., 2023) also confirmed the effectiveness of Yishen Huashi granules in reducing UAER in DKD patients.

Cordyceps sinensis preparation can reduce renal TG accumulation in DN rats by regulating the PPARα pathway, thereby reducing glomerulosclerosis, renal tubulointerstitial injury, and renal fibrosis, as well as glomerular proteinuria and renal tubular proteinuria (Zhang et al., 2022). Based on the comprehensive metabolomics and pattern recognition technology of ultra-performance liquid chromatography-mass spectrometry, it was found that Cordyceps sinensis preparations may play a role in treating DN by searching abnormal metabolic pathways (Xu et al., 2019). From the perspective of the Chinese healthcare system, Cordyceps sinensis preparations may be a cost-effective treatment option for DN in the Chinese population (He et al., 2023). Cordyceps sinensis preparations have been used in the adjuvant treatment of clinical DKD, and their combination with conventional treatment is better than conventional treatment alone in terms of protecting renal function and reducing the clinical efficacy of proteinuria, which has been clinically confirmed (Li and Xu, 2020; Yu et al., 2022; Zhang et al., 2023). A meta-analysis of 38 RCT studies involving 3,167 patients with DKD showed that Cordyceps sinensis preparations combined with ACEi/ARB significantly reduced 24-hour urinary protein compared with the ACEi/ARB alone group [standardized mean difference (SMD) = −1.99, 95% confidence interval [CI] (−2.68, *P* < 0.05; −1.31, *P* < 0.01], urinary microalbumin [mean difference (MD) = −37.41, 95% CI (−44.76, −30.06), P < 0.01], UAER [MD = −24.11, 95% CI (−30.54, *P* < 0.01), −17.68), *P* < 0.01], and UACR [SMD = 1.01, 95% CI (−1.73, −0.29), *P* < 0.01], and no significant difference was found in adverse events between the two groups (Yan et al., 2023).

Moreover, Shenshuaining (Ma et al., 2023), Fufang Xueshuantong (Tang et al., 2020), Shenyan Kangfu tablets (Wang et al., 2021; Chen et al., 2021) and Liuwei Dihuang decoction (Liao et al., 2020), which are also combined with Western medicine for the treatment of DKD and effective reduction of proteinuria, should be further confirmed by real-world research and more high-quality, large-scale, and multicenter RCT studies. Chinese patent medicine has broad development potential and an upward space for treating DKD proteinuria (Zhang et al., 2022; Huang et al., 2023; Wu et al., 2023).

Overall, this review summarizes the pharmacological mechanisms underlying albuminuria in patients with DKD (Table 1).

**TABLE 1:** Mechanisms of pharmacologic treatment of albuminuria in DKD.

Drugs	Therapeutic mechanisms for albuminuria in DKD	References
Angiotensin-converting-enzyme inhibitors/angiotensin receptor blockers	1. The glomerular pressure is decreased due to the relative relaxation of the efferent arterioles 2. The reduction of glomerular albumin leakage is achieved by decreasing the surface area of the filtration barrier through glomerular contraction 3. Exerting an anti-inflammatory effect and decreasing proteinuria	1. Lovshin et al. (2018) 2. Nistala et al. (2021) 3. Cantero-Navarro et al. (2021); Crowley et al. (2024)
SGLT2 inhibitors	1. Block the reabsorption of sodium and glucose by SGLT2, and condense the afferent arterioles 2. Increase podocyte autophagy, reduce podocyte lipid content, and protect podocyte 3. Inhibition of YAP/TAZ activation and CYP4A/20-HETE signaling to alleviate tubulointerstitial fibrosis or glomerulosclerosis	1. Muskiet et al. (2019); Warren et al. (2019); Hartman et al. (2020); Kogot-Levin et al. (2020); Wang et al. (2021a); González-Albarrán et al. (2022); Kim and Kim (2022); Salvatore et al. (2022) 2. DeFronzo et al. (2021); Chen et al. (2022); Durcan et al. (2022) 3. Dia et al. (2023); Feng et al. (2023)
Nonsteroidal mineralocorticoid receptor antagonists	1. Mitigate the pathological alterations of glomerular and tubular injury by attenuating renal inflammation and fibrosis 2. Alleviate intraglomerular pressure through reduction of glomerular hyperfiltration	1. Agarwal et al. (2021); Barrera-Chimal et al. (2022a) 2. Barrera-Chimal et al. (2022a); Kim et al. (2023); Ortiz et al. (2023)
Endothelin receptor antagonists	It binds to endothelin −1 to protect the glomerular filtration barrier and reverse glomerular hyperfiltration. Protect the renal tubular epithelial cells, relieve renal tubular interstitial fibrosis.	Allison (2016), Garsen et al. (2016), Dolinina et al. (2019)
Keluoxin capsule	1. Improve glucolipid metabolism, improve microcirculation, improve blood flow dynamics 2. Lower levels of oxidative stress and inflammation factors 3. Regulation of podocyte autophagy	1. Chunxue et al. (2023); L et al. (2000) 2. Kong et al. (2022); Deng et al. (2023); Huimin et al. (2023); XM et al. (2023) 3. Yang et al. (2021)
Huangkui capsule	1. Inhibition of JAK2/STAT3 and PI3K/Akt pathways protects podocytes 2. Modulating gut microbiota to improve metabolite levels in DN. 3. Blocking Klotho/TGF - beta 1/p38MAPK, TLR4/nf-kappa B signaling pathway, inhibiting the expression of renal fibrosis-related proteins.	1. Zhao et al. (2023) 2. Shi et al. (2023) 3. Han et al. (2019); Gu et al. (2021)
Tripterygium glycosides	It can promote the decomposition of renal triglyceride and improve the damaged renal function	Zhang et al. (2022a)
Niaoduqing granule	1. Inhibition of the AGR/RAGE pathway activity and the expression of related inflammatory factors can prevent podocyte injury. 2. Regulate p38MAPK/nf-kappa B signaling pathway to ameliorate renal fibrosis.	1. Fang et al. (2023) 2. Li et al. (2022b)
Yishen Huashi granule	1. Ameliorate podocyte injury induced by macrophage-derived exosomes 2. Regulation of intestinal flora and serum metabolites, and improvement expression of metabolism-related mRNA.	1. Liang et al. (2022) 2. Han et al. (2023)
Cordyceps sinensis preparation	1. Regulation of PPARα pathway to reduce renal triglyceride accumulation 2. Retrieval of abnormal metabolic pathways	1. Zhang et al. (2022c) 2. Xu et al. (2019)

### 5.6 Targeted precision therapy

Although these drugs have shown encouraging efficacy in treating DKD proteinuria, some patients continue to experience uremia. Targeted drug delivery strategy is a rapidly developed method in which the specific bio-actives are transported with carrier system to the predetermined organ or cell. It enables the therapeutic agents specifically transfer and accumulate at the diseased sites and increases the local concentration of drugs and minimizes the side effects (Chen et al., 2023). Increasing evidence has shown that targeted drug delivery strategies, such as macromolecular carriers, nanoparticles, and liposomes, can improve drug efficacy and reduce adverse side effects, which may become a new milestone in treating DKD (Chen et al., 2023). HDAC4 is involved in podocyte injury in DKD, and HDAC4 siRNA shows good therapeutic prospects for DKD. DTsiANp/HDAC4 can deliver HDAC4 siRNA to the podocytes of DKD rats, and a 4-week intervention can significantly reduce UAER and glomerulosclerosis (Raval et al., 2019). Targeted renal delivery of gold nanoparticles with a diameter of 50 nm can reduce UAER, glomerular basement membrane thickness, and foot process width in DKD rats (Alomari et al., 2020). As an antioxidant, Coenzyme Q10 may be a promising treatment option for early-stage DKD. However, its low water solubility and nonspecific distribution limit its clinical application. Therefore, liposomes containing CoQ10 combined with ultrasound microbubbles were injected into DKD rats to target the kidneys and improve proteinuria and oxidative stress markers (Yue et al., 2017). The above studies are still in the preclinical stage; however, as the research on the pathogenesis of DKD advances, targeted precision therapy will be explored for clinical applications.

## 6 Polydrug therapy for proteinuria in patients with T2DKD

A long history of care has discouraged the use of polypharmacy (more than five different drugs) in patients with diabetes because of the risk of adverse drug–drug interactions (Guillot et al., 2020). However, patients with diabetes mellitus frequently experience various complications and comorbidities. Multidrug treatment for lowering blood glucose, blood pressure, and lipids has been widely accepted because its efficacy has been confirmed, and the doses used are lower than a single drug dose, with fewer side effects.

Many clinical studies have shown that controlling proteinuria in patients with DKD can delay the progression of cardiovascular disease and CKD and reduce the risk of death. ACEi or ARB are recommended as the first treatment choice for patients with T2DKD, regardless of whether they are complicated with microalbuminuria or macroalbuminuria (2014; Rossing et al., 2022a). However, a combination of ACEi and ARB is not recommended, and ACEi/ARB is not recommended for the primary prevention of DKD in patients with diabetes with normal blood pressure and proteinuria (de Boer et al., 2022). Conversely, the Scr level at which RAS should be contraindicated remains controversial (National Kidney Foundation, 2012). Non-dihydropyridine calcium channel blockers, such as verapamil, can stabilize or even reduce the level of urinary protein (Steuber et al., 2019) and can be used as the first combination of ACEi/ARB drugs when blood pressure is not well controlled (Weber et al., 2010). If a beta-blocker is combined, a vasodilator beta-blocker, including Carvedilol or Nebivolol, may be preferred because non-vasodilator beta-blockers accelerate albuminuria progression (Bell and Goncalves, 2021; Bell, 2022).

In patients with DKD, SGLT2i or MRA can be added when the target urinary albumin levels are not achieved with RAS blockade (Bell, 2022). SGLT2i can effectively reduce urinary protein levels in both micro-and macroalbuminuria (Piperidou et al., 2019). Finerenone combined with an ACRI/ARB is effective in reducing urinary protein levels in patients with T2DKD (Bakris et al., 2015), and no clear evidence of an increased risk of hyperkalemia or acute kidney injury exists. The FIDELIO-DKD and CREDENCE trials showed similar cardiorenal benefits (Agarwal et al., 2022b). Therefore, triple oral therapy with a RAS blocker, an SGLT2i, and an MRA should be considered if the urinary albumin level remains high. To date, no conclusive evidence exists that triple therapy is effective in treating DKD; however, a retrospective report from a prospective study showed that the addition of finerenone can further reduce proteinuria and delay the decline in renal function in patients with DKD who have already received RAS inhibitors, and a small number of individuals in this group also received SGLT2i (McGill, 2021). A study with UACR as the endpoint (CONFIDENCE) was designed to inform the scientific and clinical community whether the triple combination of RASi, finerenone, and empagliflozin has additional benefit in patients with CKD and T2D (Green et al., 2023). The future advancements of this study are eagerly anticipated. Patients with advanced DKD should consider the risk of undertreatment. Therefore, whether triple therapy can be used in patients with diabetes with micro-and macroalbuminuria in the future requires further high-quality, medical-based evidence.

In clinical practice, there has been no experience with the combination of these drugs and ERA. Theoretically, SGLT2i can reduce sodium and water retention in ERAs by constricting afferent arterioles. ERA dilates efferent arterioles and synergistically reduces glomerular hyperfiltration while complementing SGLT2i. However, an experiment in male db/db mice without kidney resection showed no significant differences in urinary albumin excretion, and histopathological changes were observed in the SGLT2i and ET-A antagonist groups compared with the SGLT2i group alone (Stuart et al., 2022).

## 7 Conclusion and prospect

DKD is a heterogeneous disease characterized by multiple factors and complex phenotypes. Pathophysiological differences among patients can lead to individual differences in drug responses. Therefore, large-scale prospective trials based on individual drug responses and phenotypic characteristics are required to guide precise treatments. Optimal combinations of new therapies can further delay DKD progression. Real-world studies can clarify which combination is beneficial for cardiac and renal outcomes in patients with T2DKD while being well tolerated, safe, easy to manage, and cost-effective. Simultaneously, to better understand the mechanism of DKD, particularly T2DKD, basic research should be intensified, especially based on the metabolic pathways and metabolites of renal cells in chronic high-glucose environments, and focus on the metabolic and hemodynamic pathways in the DKD environment. Furthermore, targeting the AGE/RAGR axis involves focusing on new targets for renoprotective intervention, such as AGE inhibitors pyridoxamine and glyoxalase-1, an enzyme that degrades the AGE precursor methylglyoxaldehyde (Batu Demir and Cooper, 2016). Therefore, attention should be paid to the kinase cascade initiated by metabolite reactive oxygen species and the transcription factors involved in the reaction. Finally, the targeted drug delivery strategy for DKD is still in its early stages, and there are limitations regarding safety, stability, and clinical efficacy. The selection of the best kidney-targeting strategy is expected to provide a new treatment approach for DKD.
